# Population-based outreach versus care as usual to prevent suicide attempt: study protocol for a randomized controlled trial

**DOI:** 10.1186/s13063-016-1566-z

**Published:** 2016-09-15

**Authors:** Gregory E. Simon, Arne Beck, Rebecca Rossom, Julie Richards, Beth Kirlin, Deborah King, Lisa Shulman, Evette J. Ludman, Robert Penfold, Susan M. Shortreed, Ursula Whiteside

**Affiliations:** 1 Group Health Research Institute, 1730 Minor Ave. #1600, Seattle, WA 98101 USA; 2 Institute for Health Research, Kaiser Permanente Colorado, Denver, CO USA; 3 HealthPartners Institute, Minneapolis, MN USA

**Keywords:** Suicide, Prevention, Pragmatic trial, Care management, Consent waiver

## Abstract

**Background:**

Suicide remains the 10th-ranked most frequent cause of death in the United States, accounting for over 40,000 deaths per year. Nonfatal suicide attempts lead to over 200,000 hospitalizations and 600,000 emergency department visits annually. Recent evidence indicates that responses to the commonly used Patient Health Questionnaire (PHQ9) can identify outpatients who are at risk of suicide attempt and suicide death and that specific psychotherapy or Care Management programs can prevent suicide attempts in high-risk patients. Motivated by these developments, the NIMH-funded Mental Health Research Network has undertaken a multisite trial of two outreach programs to prevent suicide attempts among outpatients identified by routinely administered PHQ9 questionnaires.

**Methods/design:**

Outpatients who are at risk of suicide attempt are automatically identified using data from electronic health records (EHRs). Following a modified Zelen design, all those identified are assigned to continued usual care (i.e., no contact) or to be offered one of two population-based outreach programs. A Care Management intervention includes systematic outreach to assess suicide risk, EHR-based tools to implement risk-based care pathways, and care management to facilitate recommended follow-up. A Skills Training intervention includes interactive online training in Dialectical Behavior Therapy skills, supported by reminder and reinforcement messages from a skills coach. Each intervention supplements, rather than replaces, usual care; participants may receive any other services normally available. Interventions are delivered primarily by secure messaging through EHR patient portals. Suicide attempts and deaths following randomization are identified using state vital statistics data and health system EHR and insurance claim data. Primary evaluation will compare risk of suicide attempt or death over 18 months according to the initial assignment, regardless of intervention participation. Recruitment is underway in three health systems (Group Health Cooperative, HealthPartners, and Kaiser Permanente Colorado). Over 2500 participants have been randomized as of 1 March 2016, with enrollment averaging approximately 100 per week.

**Discussion:**

Assessing the effectiveness of population-based suicide prevention requires adherence to the principles of pragmatic trials: population-based enrollment, accepting variable treatment participation, assessing outcomes using health record data, and analyses based on intent-to-treat.

**Trial registration:**

ClinicalTrials.gov registration #NCT02326883, registered on 23 December 2014.

## Background

Suicide remains the 10th-ranked most frequent cause of death in the United States, accounting for over 40,000 deaths per year [1]. Nonfatal suicide attempts lead to over 200,000 hospitalizations and 600,000 emergency department visits each year [2, 3]. In contrast with other common causes of death, suicide mortality has not decreased over the last 25 years.

While prevention of suicide attempts and suicide death is a public health priority, existing evidence does not clearly support selective or secondary prevention programs. In 2013, the US Preventive Services Task Force found insufficient evidence to support the benefits of screening for suicide risk in general medical outpatients [4]. That review found insufficient evidence both for the accuracy of screening tests and for the effectiveness of interventions in those identified by screening.

More recent evidence indicates that responses to the commonly used Patient Health Questionnaire (PHQ9) can identify outpatients who are at increased risk of suicide attempt and suicide death [5, 6]. Patients reporting frequent thoughts of death or self-injury (the ninth item of the PHQ9) show a sustained increase in risk, with cumulative hazard approaching 4 % over 12 months. Reflecting this new evidence, the Joint Commission recently issued a Sentinel Event Alert [7] regarding detection of suicidal ideation in health care settings.

In addition, growing evidence supports the effectiveness of tertiary or indicated prevention interventions for high-risk patients. Structured psychotherapy emphasizing specific behavioral and cognitive skills has been proven to reduce risk among people who have made recent suicide attempts [8–10]. Outreach and Care Management programs appear to reduce risk among people who have made recent suicide attempts or high-risk patients treated in mental health specialty clinics [11]. This evidence for tertiary prevention suggests that similar interventions could reduce risk in the broader (secondary prevention) population of patients who are experiencing frequent suicidal ideation.

Motivated by these developments, the National Institute of Mental Health-funded Mental Health Research Network has undertaken a multisite trial of two population-based programs to prevent suicide attempts among outpatients identified by routinely administered depression questionnaires. Both programs include systematic outreach and regular supportive contact. One focuses on risk assessment and care management [11], while the other includes online training in specific skills from Dialectical Behavior Therapy (DBT) [12]. This pragmatic trial will examine whether either program can reduce long-term risk compared to care as usual.

## Methods

### Overview

At participating health systems, outpatients who are at increased risk of suicide attempt are identified using data from electronic health records (EHRs). Following a modified Zelen [13–15] design, all those identified are automatically assigned to continue in usual care (i.e., no contact) or to be offered one of two population-based prevention programs:

- A Care Management intervention including: systematic outreach to assess risk of suicidal behavior, EHR-based tools to implement risk-based care pathways, and care management to facilitate and monitor recommended follow-up care
- A Skills Training intervention including: interactive online training in DBT skills [12], supported by reminder and reinforcement messages from a skills coach

Each intervention is intended to supplement, rather than replace, usual care provided by specialty mental health or primary care providers. Participants in all three treatment groups are free to receive any other services that are normally available, including pharmacotherapy, individual or group psychotherapy, or inpatient care. Intervention services are delivered primarily by online secure messaging through EHR patient portals [16, 17]. Nonfatal and fatal suicide attempts following randomization are identified using state vital statistics data and diagnoses of self-inflicted injury from health system clinical and insurance claim records [18, 19]. Primary evaluation will compare risk of first suicide attempt (nonfatal or fatal) over the 18 months following randomization. Groups will be compared according to initial treatment assignment, regardless of level of participation in either intervention program.

### Study settings

The study sites include three members of the Mental Health Research Network: Group Health Cooperative, HealthPartners, and Kaiser Permanente of Colorado. These health systems provide general medical and mental health specialty care as well as insurance coverage to defined member/patient populations. Patients served are representative of each health system’s geographic service area in terms of race, ethnicity, educational attainment, and household income.

**Fig. 1:**
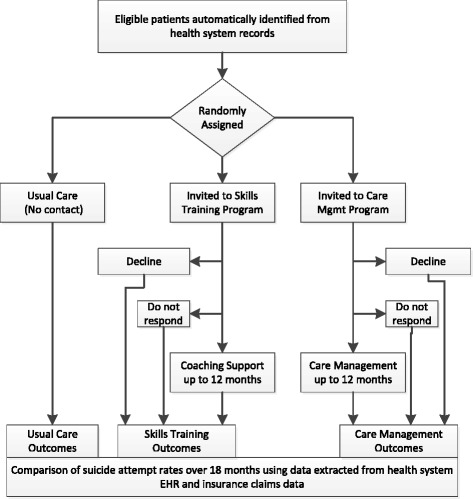
Trial flow chart

All three participating health care systems recommend the routine use of the PHQ9 depression questionnaire [20] at all mental health specialty visits and all primary care visits for treatment of depression [21]. All three sites participated in previous research [5] demonstrating that the response to item 9 of the PHQ9 (regarding thoughts of death or self-harm) predicts markedly elevated risk of suicide attempt over the following 18 months (Fig. 1).

### Eligibility

Eligibility criteria for automatic inclusion in the trial include:

- Completion of a PHQ9 questionnaire [20] at an outpatient mental health or general medical visit
- Age 18 years or older on the visit date
- Response of “more than half the days” or “nearly every day” to item 9 of the PHQ9
- Use of EHR patient portal secure messaging during the prior year
- Currently enrolled in participating health system insurance plan (to ensure adequate ascertainment of subsequent suicide attempts)

Exclusion criteria include:

- Recorded diagnosis of dementia or developmental delay in the previous 2 years
- Limited English proficiency (as indicated by “need for interpreter” recorded in the EHRs)
- Previous request to be excluded from research invitations
- Already enrolled and randomized via a previous visit

### Enrollment and randomization

Each week, EHR and insurance claim databases at each study site are used to identify all patients who meet eligibility criteria during the previous week. Immediately after sampling, all eligible patients are randomly assigned in equal proportions (1/3:1/3:1/3) to continue in usual care or to be offered one of the two intervention programs. At each site, randomization occurs automatically within the sampling computer program, stratified by eligible PHQ9 response (“more than half the days” or “nearly every day”) and site. A computer-generated concealed allocation table at each site provides randomly generated assignments in block sizes of either six or nine.

### Invitation and consent

Participants assigned to the Care Management intervention receive an initial invitation message from the study care manager via the EHR-based online secure messaging system [16, 17]. This invitation includes a brief description of the Care Management program and abbreviated information regarding required elements of informed consent (study purpose, study procedures, potential risks or harms, and right to refuse or withdraw at any time). Each participant can decline participation by replying to the invitation message or can consent to receive intervention services by either replying to the message or returning the attached risk assessment questionnaire. Participants who neither decline nor consent receive a reminder (either by online message or telephone message) after 1 week. Participants who neither decline nor consent after that reminder message receive a second invitation message (with possible reminder) 4 weeks later and may receive a third invitation message after an additional 4 weeks. Those who decline at any point are not contacted again. Those who do not respond after three cycles of invitation are not contacted again.

The invitation and consent process for participants assigned to the Skills Training program parallels that for the Care Management program: an invitation message including abbreviated informed consent information and up to three cycles of invitation and reminder for those not responding. A participant can consent to receive intervention services by replying to an invitation message or by making an initial visit to the online intervention program. Those who decline at any point are not contacted again. Those who do not respond after three cycles of invitation are not contacted again.

Participants assigned to continue in usual care are never contacted by study staff. Providers are not notified regarding participants’ assignment to continued usual care.

### Care Management intervention

#### Rationale

Following the design of previous Care Management interventions [17], including the Perfect Depression Care program at Henry Ford Health System [11], this program aims to reduce risk of suicide attempt by monitoring and maintaining engagement in effective mental health treatment.

#### Assessment tools

In collaboration with developers of the Columbia Suicide Severity Rating Scale [22] (CSSRS), study investigators developed a simplified CSSRS for self-report administration via online secure messaging. This abbreviated CSSRS provides a 6-point ordinal rating of current suicide risk based on frequency and intensity of suicidal thoughts, presence and specificity of suicidal plans, and clarity of suicidal intent during the last week. For example, a score of 0 would indicate no recent thoughts of self-injury or suicide, a score of 3 would indicate suicidal ideation with some recent thoughts about specific means, and a score of 5 would indicate a current and specific suicidal plan.

#### Follow-up algorithms

In collaboration with health system stakeholders (see below), investigators developed rules for risk-based care pathways specifying appropriate level of care, minimum standards for follow-up visit intervals and timing of outreach messages. For example: a CSSRS score of 1 would lead to a recommendation for follow-up within 1 month (sooner if clinically appropriate) in either primary care or specialty mental health care, a score of 3 would lead to a recommendation for follow-up in specialty mental health care within 2 weeks, and a score of 5 would lead to a recommendation for specialty mental health follow-up within two business days (or sooner as clinically indicated).

#### Care manager role

At each site, one or more care managers are responsible for:

- Initial and follow-up invitations to all participants assigned to be offered Care Management
- Periodic outreach to assess current risk (using an online version of the CSSRS)
- Application of follow-up algorithms, supported by informatics tools (see below)
- Regular feedback to treating providers regarding risk assessments and follow-up plans
- As-needed communication with participants and providers to facilitate follow-up care

Care managers communicate with participants primarily by online secure messaging through EHR patient portals, but may communicate by telephone as needed. Care managers are expected to consider individual patients’ clinical circumstances and providers’ treatment plans when applying algorithms regarding outreach and visit frequency. At all sites, care managers are Master’s-prepared mental health clinicians.

#### Informatics tools

Intervention delivery is supported by existing functions of health system EHRs:

- Online patient-provider secure messaging via the EHR patient portal [16, 17] for invitation and outreach to participants
- Online administration of structured questionnaires such as the CSSRS
- Secure provider-to-provider messaging for care managers’ communications with primary care and mental health specialty providers
- Population management and reporting tools to apply follow-up algorithms and deliver algorithm-based recommendations to care managers regarding outreach and follow-up

#### Engagement with health system stakeholders

During the pilot phase, regular meetings with clinical leaders from all sites (representing both primary care and mental health specialty care) developed consensus regarding content and workflow of the Care Management program, including:

- Language for outreach messages
- Recommended follow-up intervals
- Criteria for referral from primary care to mental health specialty care
- Processes for communicating with primary care and mental health specialty providers
- Procedures for urgent assessment and referral

#### Training and supervision

Training of care managers across sites was conducted by videoconference and teleconference, led by clinical investigators from the Group Health site. Initial training included:

- 6 h of clinical training regarding suicide risk assessment
- 2 h of general orientation to project aims and procedures
- 6 h of specific training regarding care management aims, tools, and procedures

Ongoing teleconference supervision for all care managers is led by clinical investigators from the Group Health site. Supervision meetings were scheduled weekly for 6 months and twice monthly thereafter. Consistent with the principles of pragmatic trials [23], no detailed monitoring of intervention fidelity (e.g., review of content of online messaging or phone contacts) is conducted.

### Skills Training intervention

#### Rationale

Following the content and structure of proven Dialectical Behavior Therapy (DBT) treatments [8, 12], this program aimed to reduce risk of suicide attempt through training in specific DBT skills shown to mediate the beneficial effects of traditional in-person DBT [12].

#### Specific skills content

The online program and coaching support focuses on four specific skills:

- Mindfulness – Introduction to mindfulness skills, emphasizing nonjudgmental observation
- Mindfulness of Current Emotion – Nonjudgmental observation of sensations associated with difficult or painful emotions
- Opposite Action – Acting in opposition to urges associated with painful or difficult emotions
- Paced Breathing – Use of breathing techniques to manage overwhelming emotions or crises

#### Adaptation for online delivery

The web-based interactive program includes an introductory section, personal profiles of team members (including coaches and contributing peer experts described below), and four skill modules (one for each DBT skill listed above). Each module includes:

- A brief video introduction to the skill concept
- A longer teaching video describing the skill, including in vivo practice
- Example videos of peers (see below) describing use of the skill in daily life
- Interactive exercises for use during the online session
- Customizable worksheets to support between-session practice

Each participant is free to visit skills modules in any order and use the program at any pace, returning as frequently as desired. Coaches send reinforcement and outreach messages (see below) to encourage regular use.

#### Skills coach role

At each site, one or more skills coaches is responsible for:

- Initial and follow-up invitations to all participants assigned to be offered Skills Training
- For participants visiting the online program, messages to reinforce use of the program and practice of specific skills
- For participants not visiting the program, periodic outreach messages to encourage return visits
- As-needed communication with treating providers regarding participants’ progress

Skills coaches communicate with participants primarily by online secure messaging through EHR patient portals, but may communicate by telephone as needed. At all sites, skills coaches are Master’s-prepared mental health clinicians.

#### Informatics tools

The online program is delivered through the DatStat survey platform (DatStat Inc., Seattle, WA, USA). This platform supports secure access, detailed tracking of participant activity, and participant-level reports to guide the timing and content of coaches’ reinforcement and reminder messages. Participants access the online program via secure personalized links embedded in messages from skills coaches.

#### Engagement with patient stakeholders

Peer experts (people with lived experience of suicidal ideation and suicide attempts) were essential collaborators in the development of the Skills Training intervention and continue to support intervention delivery [24]. Design of outreach messages and content of the online program were informed by anonymous online surveys and focus group interviews with peer experts [24]. People with lived experience contributed video descriptions of the use of DBT skills, and continue to contribute to development of training and support materials for skills coaches.

#### Training and supervision

Training of skills coaches was conducted by videoconference and teleconference, led by clinical investigators from the Group Health site. Initial training included:

- 6 h of clinical training regarding suicide risk assessment
- 2 h of general orientation to project aims and procedures
- 6 h of specific training regarding skills coaching aims, tools, and procedures

Ongoing teleconference supervision for all skills coaches is led by clinical investigators from the Group Health site. Supervision meetings were scheduled weekly for 6 months and twice monthly thereafter. Consistent with the principles of pragmatic trials [23], no detailed monitoring of intervention fidelity (e.g., review of content of online messaging or phone contacts) is conducted.

### Outcome definitions

The primary study outcome is the time to first suicide attempt following randomization. Fatal suicide attempts will be identified by death certificate diagnoses of self-inflicted injury or poisoning. All three study sites routinely link membership files to state vital record data to ascertain cause of death for all enrolled members. Nonfatal suicide attempts will be identified from EHRs (for care delivered by participating health systems) and insurance claim data (for care received outside of participating health systems) using three criteria:

- Any outpatient or inpatient diagnosis of definite self-inflicted injury or poisoning
- Any outpatient or inpatient diagnosis of possible self-inflicted injury or poisoning
- Any outpatient or inpatient diagnosis of other injury or poisoning associated with a diagnosis of suicidal ideation during the same encounter

For these three criteria, review of full-text medical records documented high positive predictive value for self-inflicted injury with suicidal intent [18, 25]. Because this validation work was completed prior to health care systems’ transition from the *International Classification of Diseases, version 9* (ICD-9) to ICD-10 diagnoses, additional work will be necessary in 2016 to revalidate outcome definitions based on ICD-10 diagnostic codes. Given that participants may seek care for self-injury at external facilities, ascertainment will include both insurance claim and EHR data, and the sample is limited to patients who are enrolled in a health system insurance plan.

### Analysis plan

Primary analyses will use the log-rank test to compare risk of diagnosed suicide attempt (defined above) over 18 months following randomization. For each intervention condition, risk among those assigned to the intervention will be compared to risk among those assigned to usual care – regardless of level of participation in either intervention. Individuals will be censored at time of health system disenrollment, death from cause other than suicide, or administratively, at 18 months following randomization. We evaluate the effect of each of the interventions compared to usual care using a log-rank test stratified by site and initial response to PHQ9 item 9 (2 versus 3). Sensitivity analyses will use weighted log-rank tests to account for a possible association between prerandomization characteristics (ascertained from EHRs) and censoring. In censoring weights, we will include sex, age group (18–29, 30–64, and 65 or more), race/ethnicity (Black American, Asian American, Hispanic, other), and visit type at which the initial PHQ9 questionnaire was completed (primary care versus mental health specialty).

### Sample size

Original sample size estimates were based on previous research [5] suggesting an approximately 4 % risk of suicide attempt over 18 months among those meeting study eligibility criteria. Consultation with health system stakeholders indicated that implementation of a systematic outreach program was unlikely unless a program could be expected to reduce that risk to 3 % (relative risk reduction of 25 %). We used PASS software [26] to estimate the sample size required for a log-rank test [27] with 90 % power to compare the survival curves assuming a 3.8 % risk over 18 months in the comparison group and a 25 % risk reduction in the intervention group. We assumed 2 % disenrollment rate each month, resulting in approximately 25 % censorship over the 18 months of follow-up. The primary comparison for this trial is each of the interventions compared to usual care; we use a Bonferroni correction to account for the two tests in our primary analysis. Assuming a two-sided log-rank test, with a type-1 error rate of 0.025 and 90 %, we plan on enrolling 6500 patients per arm (total *n* = 19,500).

### Enrollment progress

The trial was funded through the NIH Health Care Systems Collaboratory as one of the initial Pragmatic Clinical Trials Demonstration Projects [28]. Following a pilot phase to validate outcome definitions and to demonstrate the feasibility and acceptability of the intervention programs, participant enrollment and randomization began at the Group Health site in March 2015, expanding to three sites in July 2015. Approximately 4000 participants have been enrolled and randomized as of 1 July 2016. Approximately 100 participants are enrolled and randomized each week, and that rate is expected to increase to approximately 150 in the fall of 2016.

### Ethical and regulatory approval

Study design and procedures were reviewed and approved by Institutional Review Boards at all three health system. That review process addressed several issues common to pragmatic trials of prevention interventions.

### Waiver of informed consent

Limiting a randomized trial of outreach to those who actively consent to receive outreach would yield a result of questionable validity and generalizability. Consequently, a modified Zelen design [13–15], randomizing all eligible patients without first obtaining consent, is necessary for valid test of the study question. This design, however, requires a waiver of the usual requirement for informed consent prior to enrollment or randomization. While it is not practicable to obtain informed consent prior to randomization, it is practicable to provide appropriate information to participants at the time intervention services are offered. As described above, invitation messages to participants assigned to either intervention include a brief description of the study purpose, study procedures, potential risks, and the right to decline participation. This design, therefore, includes a waiver of consent for enrollment/randomization and a modified consent procedure for receipt of intervention services.

### Defining minimal risk

Current regulations regarding protection of human research participants allow waiver of consent for research involving no more than minimal risk. Our proposal to waive the requirement for informed consent in patients who are at risk of suicide attempt led to extensive discussions with health system Institutional Review Boards, leadership of the NIH Healthcare Systems Research Collaboratory, and the federal Office for Human Research Protections [29]. Those discussions helped to clarify four issues:

- Research risk versus preexisting risk – Given that the trial enrolls patients who are at risk of suicide attempt, we encountered concern that study procedures could not be classified as having minimal risk. To address this concern, we relied on regulatory guidance distinguishing between preexisting risk due to a research participant’s health state (i.e., increased risk of suicide attempt) and incremental risk created by study procedures. This distinction led to the appreciation of this trial as evaluating minimal-risk interventions in a high-risk population
- Risk of assignment to continued usual care – We also encountered concern regarding the ethical acceptability of randomly assigning patients who are at risk of suicide attempt to a usual care control condition. We clarified that a participant assigned to usual care will, by definition, receive the same treatment that she or he would have received if the study were not occurring
- Risk of assignment to offer of intervention programs – We also encountered concern that assignment to either intervention group might increase risk. Both intervention programs are based on effective interventions and are intended to reduce risk of suicide attempt. Participants are free to receive any other services that are normally available. Nevertheless, it is possible that some participants will experience negative effects from either program. We addressed this concern by clarifying that participants are assigned to the offer of an intervention, with no obligation to participate. Invitation messages clearly identify intervention programs as research activities, make no promise of benefit, and advise that participation is completely voluntary
- Intrusiveness or invasion of privacy – Different stakeholders expressed concern regarding both inappropriate intrusiveness of repeated outreach and inadequate vigor of outreach given the known increased risk of suicide attempt. In consultation with peer experts, we designed the outreach strategy described above, including up to three cycles of invitation, as a reasonable compromise between these two concerns.

At all participating health systems, notices regarding privacy practices specifically advise members regarding the use of health records for research. Members who have previously requested exclusion from research contact are excluded from the study sample.

### Monitoring for adverse events

In most clinical trials of mental health treatments, a suicide attempt would be considered a serious adverse event, subject to immediate reporting and review to determine the “relatedness” of an individual suicide attempt to study participation. This traditional approach was clearly not appropriate for a large-scale trial of population-based prevention programs [30]. First, record data regarding suicide attempts may not be available for 3 months or more following an event. Second, several hundred suicide attempts are expected to occur among study participants, and review of individual events could not determine causal relationship to study participation. While it is possible that either intervention could paradoxically increase risk of suicide attempt, that could only be determined by comparison to suicide attempt rates in usual care (see below).

### Interim analyses of benefit or harm

We do not plan any interim analyses to evaluate benefit of the intervention programs. First, early detection of a benefit of either intervention is extremely unlikely. We project that randomization will be complete before complete outcome data are available for half of the participants. Second, early termination of randomization or intervention delivery would not offer any additional protection to current or future study participants. Premature termination would instead return all current and potential participants to care as usual.

We do, however, plan interim analyses testing for evidence of significant harm (increased risk of suicide attempt) in either intervention group compared to usual care. Clear evidence that either intervention resulted in significantly increased risk of suicide attempt would certainly warrant suspending assignment of patients to that program or suspending delivery of that program to participants already assigned. Interim analyses comparing risk of suicide attempt in each intervention group to that in usual care will be conducted three times per year, beginning 12 months after start of enrollment. Interim analyses will be reported to the National Institute of Mental Health Data and Safety Monitoring Board by the study statistician, but all other study staff will be blinded to these results.

### Data and resource sharing

A deidentified version of the analytic dataset will be made available at the time of the initial publication of primary study findings. Consistent with policies of the NIH Collaboratory, all resources (intervention materials, specifications, computer code, etc.) will be shared at or before the publication of study results.

## Discussion

This suicide prevention outreach trial addresses a practical question that is relevant to practicing clinicians or health system leaders: Will population-based outreach programs reduce risk of suicide attempt among patients identified as being at risk by routinely administered depression questionnaires? This focus on a practical or pragmatic question has several implications for trial design. Table 1 describes how specific aspects of this trial conform to the characteristics of pragmatic trials described by Thorpe [31]. Central pragmatic trial features include:

**Table 1:** PRECIS domains defining pragmatic trials

	PRECIS criteria for pragmatic trials	Design of suicide prevention outreach trial
Participants	All eligible participants enrolled, regardless of risk, responsiveness, comorbidities or past compliance	Adult health plan members reporting frequent suicidal ideation on routine depression questionnaires are automatically enrolled
Intervention condition	Interventions are highly flexible, offering providers leeway in formulation and application	Both interventions allow personalization to patients’ needs and preferences. Varying levels of participation are expected
Intervention practitioners	Interventions are applied by the full range of practitioners in the full range of settings with only ordinary attention to dose and side effects	Intervention clinicians will be recruited from existing local workforces. Each site will be responsible for selection and supervision of clinicians (using standard quality control tools)
Comparison condition	“Usual practice” (or the best alternative), offering practitioners considerable leeway in application	Each prevention program will be compared to usual care
Comparison practitioners	The control intervention is applied by the full range of clinicians in the full range of settings, with only ordinary attention to training, experience, and performance	Usual care will be provided by real-world providers (mental health and general medical clinicians) under usual practice conditions – with no additional training or supervision
Follow-up assessments	There are no research assessments; administrative databases are searched for outcomes	All outcome data are collected from EHR, insurance claim data, and death certificate data
Outcome definition	The primary outcome is objectively measured, meaningful to study participants, and does not depend on central adjudication	Primary and secondary outcomes are defined by specific ICD-9/ICD-10 diagnosis codes – no clinical assessment is required
Intervention compliance	There are no special strategies to improve compliance, and compliance is unobtrusively measured	Patients assigned to interventions are free to participate (or not participate) at any level. Participation or compliance is assessed passively using EHRs and online intervention databases
Practitioner adherence	There are no special strategies to maintain practitioner adherence, and adherence is unobtrusively measured	Care managers and skills coaches work independently at each site, but receive initial training and regular supervision from study investigators
Primary comparison	The analysis includes all patients regardless of compliance, eligibility, or others	All outcomes will be analyzed according to initial assignment – regardless of intervention participation or compliance

*EHR* electronic health record, *ICD* International Classification of Diseases

- Population-based enrollment – If we hope to inform policy or implementation decisions by health system leaders, then it is necessary to evaluate program effectiveness in the full population of those to whom the program would eventually be offered
- Allowing variable participation or compliance – Restricting enrollment to those willing to participate in outreach or prevention programs would not allow a valid assessment of program effectiveness. Low participation or high rates of dropout should be considered essential indicators of effectiveness rather than threats to internal validity
- Analysis by intent-to-treat – A valid evaluation of prevention program effectiveness must examine risk among all those offered the prevention service, rather than those who accept or participate. Any “as treated” or “completers” analysis (limited to those who participate in prevention services) would certainly be biased

Our trial differs from a purely pragmatic design in one aspect: the training and supervision of clinical staff delivering the prevention interventions. All care managers and skills coaches complete approximately 14 h of initial training followed by weekly or bi-weekly supervision teleconferences. This training and supervision was necessary because both of these clinical roles required implementation of new clinical work processes and the use of new informatics tools. If either program is proven effective, we would recommend that any subsequent implementation include a similar level of training as well as a period of regular supervision.

Assessing the effectiveness of any population-based prevention program requires a clinical trial following the core principles of pragmatic trials: population-based enrollment, accepting variable treatment participation, assessing outcomes using health record data, and analyses based on intent-to-treat. We describe the design and implementation of such a trial, now underway in three large integrated health systems.

## Trial status

Enrollment is ongoing and is expected to be complete in early 2018.

## Abbreviations

CSSRS: Columbia Suicide Severity Rating Scale
DBT: Dialectical Behavior Therapy
EHR: Electronic health record
PHQ9: Patient Health Questionnaire depression scale
